# Screening and Selection of Native Lactic Acid Bacteria Isolated from Chilean Grapes

**DOI:** 10.3390/foods14010143

**Published:** 2025-01-06

**Authors:** Carla Vargas-Luna, Liliana Godoy, Sergio Benavides, Consuelo Ceppi de Lecco, Alejandra Urtubia, Wendy Franco

**Affiliations:** 1 Departamento de Ingeniería Química y Bioprocesos, Pontificia Universidad Católica de Chile, Santiago 6904411, Chile; cpvargasluna@gmail.com; 2 Departamento de Fruticultura y Enología, Facultad de Agronomía y Sistemas Naturales, Pontificia Universidad Católica de Chile, Santiago 6904411, Chile; liliana.godoy@uc.cl (L.G.); ceppidelecco@uc.cl (C.C.d.L.); 3 Carrera de Nutrición y Dietética, Escuela de Nutrición y Dietética, Facultad de Ciencias para el Cuidado de la Salud, Universidad San Sebastina, Campus Las Tres Pascualas, Lientur 1457, Concepción 4080871, Chile; sergio.benavides@uss.cl; 4 Departamento de Ingeniería Química Medio Ambiental, Universidad Técnico Federico Santa María, Av. 12 España 1680, Valparaíso 2390123, Chile; alejandra.urtubia@usm.cl

**Keywords:** native bacteria, starter culture, *Levilactobacillus brevis*, malolactic fermentation

## Abstract

The aim of this study was investigating the biological diversity of lactic acid bacteria isolated from Chilean grapes and identifying potential candidates for use as malolactic fermentation starter cultures. The isolated bacteria underwent a comprehensive six-stage screening process, which was mutually exclusive except for the evaluation of tyramine production and citric acid intake. This process included morphological, metabolic, fermentation yield, and resistance tests to identify promising malolactic strains. Morphological assessments led to the selection of 23 isolates, which were genetically identified as *Levilactobacillus brevis* (65% abundance) and *Leuconostoc mesenteroides* (35% abundance). Among these, eight strains exhibited low sugar metabolism rates, while three demonstrated competitive growth and malolactic activity in a synthetic medium containing 10% ethanol, outperforming a commercial strain. Low consumption was observed in the qualitative citric acid intake test, whereas a positive response was noted for tyramine production. At the conclusion of the sequential selection criteria, *Levilactobacillus brevis* BCV-46 exhibited the most favorable characteristics for potential use as a malolactic starter culture, successfully withstanding the combined stress factors of ethanol, pH, and SO_2_.

## 1. Introduction

Worldwide, *Vitis vinifera*, commonly known as the wine grape, is a crop of significant economic and cultural importance. The surfaces of grapes host diverse grapevine-associated microbial communities that collectively form the microbial composition and influence the characteristics of wine. These communities are non-randomly associated with soil, climate, topography, and human practices, all of which contribute to shaping the terroir effect [1]. Among the main microorganisms found, yeasts play a predominant role in the winemaking process, leading to alcoholic fermentation (AF). Additionally, a secondary but desirable microbial-driven process known as malolactic fermentation is also important in winemaking.

Malolactic fermentation (MLF) is a secondary fermentation driven by lactic acid bacteria (LAB) that occurs at the end of or concurrent with the AF. During MLF, L-malic acid is converted into L-lactic acid and carbon dioxide. This transformative reaction plays a crucial role in winemaking by reducing the harsh acidity of malic acid (deacidification) and enhancing sensory complexity and microbial stability. Its function is to ‘soften’ most red wines and some sparkling base and white wines [2,3,4,5]. The effects of MLF are becoming increasingly recognized due to various outcomes linked to the metabolic activities associated with the growth and development of LAB. These activities have the potential to impact the quality of wine significantly [6].

Of all the indigenous LAB, the most common species that have been found in grape must and wine are *Lactiplantilactobacillus plantarum*, *Lentilactobacillus hilgardii*, *Levilactobacillus brevis*, *Lentilactobacillus buchneri*, *Lacticaseibacillus casei*, *Lactobacillus fermentum*, *Lactobacillus collinoides*, *Fructilactobacillus fructivorans*, *Lactobacillus kunkeei*, *Lactobacillus mali*, *Lactobacillus nagelii*, *Pediococcus dannosus*, *Pediococcus parvulus*, *Leuconostoc mesenteroides*, and *Oenococcus oeni* [7,8,9,10].

*Oenococcus oeni* is the preferred species used to conduct MLF because it can overcome the combined stress factors in wine. *O. oeni* is a Gram-positive microorganism that usually grows more slowly than other LAB, but it prevails and dominates MLF due to its adaptation to the wine conditions [11]. Due to this reason, strains of *O. oeni* have been adapted as commercial starter cultures. However, inconsistent and unsuccessful results for MLF under difficult wine conditions have been reported. These results are mainly associated with the poor adaptation of starter cultures to the environments in different wine regions [3,11]. To address this issue, exploring other species of LAB for wine inoculation and selecting strains that are well adapted to wine conditions has been proposed [3].

A large number of species and subspecies of LAB, beyond *O. oeni*, remain unisolated and uncharacterized, restricting available options to address inconsistent and unsuccessful outcomes in MLF. Over the past decade, indigenous LAB distinct from *O. oeni*, such as those from the *Pediococcus* and *Lactobacillus* genera, have garnered attention for their fermentation properties that could potentially contribute to the winemaking profile [2]. For instance, Bou and Krieger (2012) reported the application of alcohol-tolerant malolactic strains belonging to these genera, demonstrating their capability to initiate and complete MLF immediately upon introduction, eliminating the necessity for a prior acclimatization period [12].

The development of new-generation indigenous starter cultures demands a multidisciplinary approach that requires technological challenges and valuable physiological traits. Among the technological challenges, indigenous LAB should display the ability to withstand wine conditions, express a malolactic activity, and be able to complete MLF successfully [13,14]. At the same time, evaluating valuable physiological traits considering flavor production, aroma complexity, and the wine’s wholesomeness in terms of biogenic amine production is of great importance [5].

At the moment, little is known about the indigenous bacterial diversity of Chilean white wines, particularly from the Casa Blanca Valley. Therefore, the objective of this study was to characterize the diversity of lactic acid bacteria in two Chilean grape varieties and to identify strains with the potential to perform as starter cultures for malolactic fermentation. For this purpose, Sauvignon Blanc and Chardonnay grapes were collected from a local vineyard in Chile. Microbial diversity was isolated using traditional microbiological techniques. The isolated strains were subjected to a series of screening tests to evaluate their ability to grow and carry out malolactic fermentation under wine-like conditions using synthetic media.

## 2. Materials and Methods

### 2.1. Sample Collection

**Figure 1:**
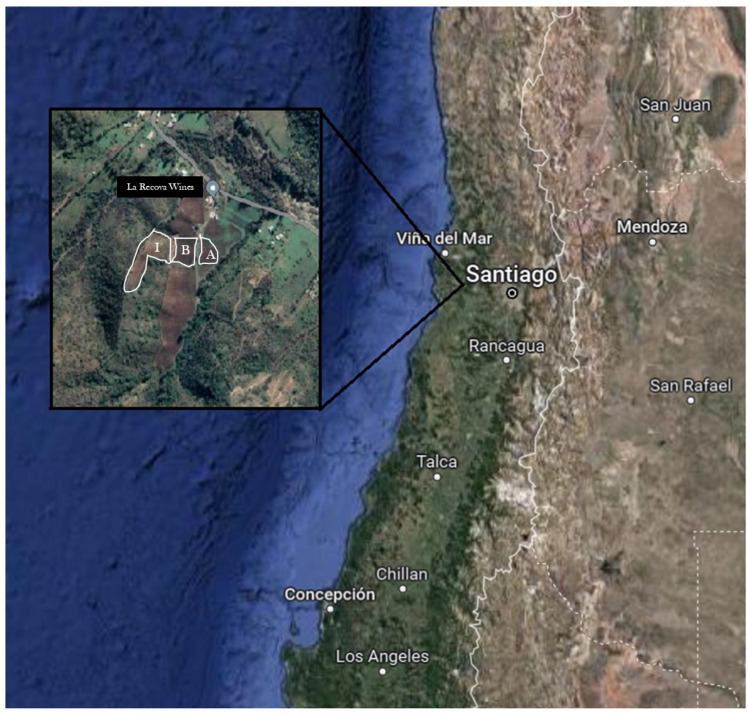
La Recova Winery location (Map data © [2023] Google).

Grape berries were collected from the La Recova Winery located in the Valparaiso Region (33°16′26.5″ S 71°31′44.7″ W, Chile, Figure 1) during the 2021 vintage season. Two grape varieties were chosen, Chardonnay and S. Blanc, and were collected from three different geographic zones (Chardonnay from zone I and S. Blanc from zones A and B), as is delimited in Figure 1. Samples for each variety were randomly hand-picked following a zig-zag pattern in triplicate and transported refrigerated to the Fermented Food Laboratory at Pontifical Catholic University of Chile (Santiago, Chile). Upon arrival, the grapes were cleaned of plant debris and leaves, then pressed to obtain the juice, which was filtered with fine gauze to eliminate large particulates.

### 2.2. Strains Isolation

The isolation and identification processes were conducted following the methodology described by Franco et al. (2021) [15]. Spontaneous fermentation was carried out without modifying the grape must. The must was left to decant for 24 h in a refrigerated chamber at 8 °C. After this time, the clarified must was extracted, avoiding clouding the must. The final must was deposited in 1-Liter Erlenmeyer flasks filled to a maximum of 80% volume. No sulfur dioxide or yeast inoculum was added to any flask. For zones A, B, and I, the initial densities were 1086 g/L, 1085 g/L, and 1086 g/L; the pH levels were 3.09, 3.12, and 3.34; and the Brix values were 21.9°, 22.4°, and 22.6°, respectively. Each flask was sealed with a lid and an airlock. The incubation was performed in triplicate at 25 °C and 150 rpm in an orbital incubator (JSR, JSS1-100C, Tokyo, Japan). Eleven days after the AF concluded, the wine was transferred to a completely filled 250 mL bottle to undergo MLF. The spontaneous MLF was completed in 48 days.

The samples were collected during AF and MLF to isolate native bacteria. Samples were serially diluted and spread-platted on de Man, Rogosa, and Sharpe agar (MRS agar, Condalab, Madrid, Spain). MRS agar plates were incubated at 28 °C for 48 h or until colonies were observed. The colonies were first grouped by morphology, and three to four independent clones (colonies) were selected based on morphology and streaked onto the same isolation media. Each colony isolated was assigned a unique identification code consisting of a combination of letters (BCV) and numbers ranging from 1 to 161.

### 2.3. Screening Criteria and Process

The screening process was designed in six stages, which were mutually exclusive except for determining tyramine production and citric acid intake, as these assays were qualitative. The strain that met the selection criteria in each stage advanced to the next stage for further evaluation

#### 2.3.1. Morphology Characterization and Identification

After isolation, a total of 161 isolates were obtained. They underwent morphology characterization through catalase reaction and Gram-staining, corresponding to the first and second stages of the selection process, respectively. All strains identified as Gram-positive and catalase-negative were selected for genetic identification. Twenty-three isolates were further identified using partial sequencing of the 16S rRNA gene, following the methodology described by Franco et al. (2021) [15]. Chromosomal DNA extraction was performed using the GeneJet Genomic DNA extraction and purification kit (Thermo Scientific, #K0772, Hampshire, UK). Each bacterium’s DNA was amplified via polymerase chain reaction (PCR) using the 27F forward primer (AGAGTTTGATCMTGGCTCAG) and 1492R reverse primer (TACGGYTACCTTGTTACGACTT) (Macrogen, Seoul, Republic of Korea). The PCR products were purified and sequenced by Macrogen (Seoul, Republic of Korea), and the sequences were analyzed using the Basic Local Alignment Search Tool http://www.ncbi.nlm.nih.gov/BLAST(accessed on 1 June 2023).

#### 2.3.2. Fermentative Metabolism

The fermentative metabolism of the identified LAB strains was evaluated using the fermentation protocol described by Reiner (2012) [16], with modifications. Briefly, green sugar broth medium (BSB) was prepared by mixing 10 g of bacteriological peptone (Millipore Merck, Darmstadt, Germany), 5 g of sodium chloride (Lider, Santiago, Chile), 0.018 g of bromocresol green (Sigma Aldrich, Saint Louis, MO, USA), and 10 g of glucose monohydrate suitable for microbiology (Merck, Darmstadt, Germany) per liter of distilled water. The BSB medium was adjusted to pH 5.5 before sterilization.

A single colony of each identified LAB strain was inoculated into 10 mL of BSB medium in sterilized tubes and thoroughly mixed. A Durham vial was placed in each tube to monitor gas production, and the tubes were capped to maintain anaerobic conditions. Incubation was carried out at 25 °C for 24 h (Zhicheng, ZDP-A2080A, Shanghai, China). Following incubation, the tubes were examined for CO_2_ production, indicated by gas accumulation in the Durham vials, and pH changes were assessed using both a bromocresol green pH indicator and a pH meter (ENZO, PL-700PV, Tainan, Taiwan). Tubes showing excessive gas production were excluded, while strains that met the required criteria were selected for further experiments. In total, eight LAB strains were chosen for continued study.

#### 2.3.3. Growth and Malolactic Activity Evaluation

Growth performance and malolactic activity expression in the presence of ethanol were evaluated for the LAB strains selected in Section 2.3.2. In order to reduce external factors that could affect the performance of the selected LAB, the methodology described by Fahimi et al. (2014) [17] was followed to prepare preculture and cultural conditions. Malolactic activity of the selected LAB strains was tested in synthetic medium M3m (MRS broth supplemented with 4 g/L malic acid (Oregon Chem, Santiago, Chile) and 2 g/L D-fructose (Merck, Darmstadt, Germany)) and pH adjusted to 3.5. The commercial culture *Lactobacillus plantarum* ML PRIME™ (Lallemand, Blagnac, France) was used as a control.

The LAB strains were cultured in 50 mL Erlenmeyer flasks under anaerobic conditions at 25 °C (JSR, JSS1-100C, Tokyo, Japan). Bacterial growth was monitored by spectrophotometry (OD) at 630 nm for 20 days (BEL Photonics, UV-W51, Monza, Italy). Samples were collected at the beginning and end of fermentation. Consumption of D-glucose, D-fructose, L-malic acid, and synthesis of L-lactic acid were determined using a Y15 photometer enzymatic autoanalyzer (BioSystems, Barcelona, Spain). L-malic acid and sugar utilization yield, and L-lactic acid production, were calculated as follows:

(1) $${\%R}_{L\to M}=\frac{L_{M}}{L_{T}}\times 100$$

where ${\%R}_{L\to M}$ is the yield of lactic acid produced from MLF (%); $L_{M}$ is the lactic acid concentration derived from malic acid consumption, determined by stoichiometric ratio (g/L); $L_{T}$ is the final concentration of lactic acid in the medium (g/L).

(2) $${\%R}_{L\to S}=\frac{L_{T}-L_{M}}{L_{T}}\times 100$$

where ${\%R}_{L\to S}$ is the yield of lactic acid produced from sugar fermentation (%).

(3) $${\%R}_{M}=\frac{M_{T}-M_{F}}{M_{T}}\times 100$$

where ${\%R}_{M}$ is the consumption yield of malic acid (%); $M_{T}$ is the initial concentration of malic acid in the medium (g/L); $M_{F}$ is the final concentrations of malic acid at the end of fermentation (g/L).

(4) $${\%R}_{G}=\frac{G_{T}-G_{F}}{G_{T}}\times 100$$

where ${\%R}_{G}$ is the consumption yield of glucose (%); $G_{T}$ is the initial concentration of glucose the medium (g/L); $G_{F}$ is the final concentrations of glucose at the end of fermentation (g/L).

(5) $${\%R}_{F}=\frac{F_{T}-F_{F}}{F_{T}}\times 100$$

where ${\%R}_{F}$ is the consumption yield of fructose; $F_{T}$ is the initial concentration of fructose in the medium (g/L); $F_{F}$ is the final concentrations of fructose at the end of fermentation (g/L).

This screening allowed us to select those strains with the highest malolactic activity for further investigation. At the end, three LAB strains were selected.

#### 2.3.4. Tolerance Assay

The tolerance to different pH levels, concentrations of SO_2_, and amounts of ethanol were tested in the M3m medium. The parameters established for the tolerance assay were based on the methodology described by Sun et al. (2016), with minimal modifications [18]. The ethanol levels were adjusted to 11.5% to 13.5%. A combined factor approach was used to test for the LAB strains selected in Section 2.3.3, as described in Table 1. Bacterial growth was followed by spectrophotometry (OD) at 630 nm (BEL Photonics, UV-W51, Monza, Italy).

#### 2.3.5. Determination of Tyramine Production and Citric Acid Intake

Tyramine production and citric acid intake were evaluated using modified solid agar, which distinguished between positive and negative tyramine production and citric acid intake based on color changes. Tyramine production was assessed following the method described by Maijala (1993) [19], employing tyrosine (Your Sups, London, UK) as the only amino acid in the media. The plates were examined for a clear halo and colony differentiation after 72 h of anaerobic incubation at 28 °C (Memmert, 30-750, Schwabach, Germany). LAB strains were classified as positive for tyramine production when a clear halo was identified. Citric acid consumption was determined using the method described by Kempler and McKay (1980) [20], using ammonium iron citrate (Sigma Aldrich, Saint Louis, MO, USA). Citric acid plates were incubated anaerobically for 24 h at 28 °C, and positive citric acid intake was indicated by the presence of blue colonies.

### 2.4. Statistical Analysis

The assays were done in triplicate and two independent runs (*n* = 6). Data were analyzed with Analysis of Variance (ANOVA), and the Tukey test was applied to determine significant differences at a significance level of *p* < 0.05. The data processing was conducted using the OriginPro version 2021 statistical package for Windows (OriginLab Corporation, Northampton, MA, USA).

## 3. Results

A six-stage screening process was designed to evaluate the fermentation potential and the ability to be a possible starter culture for MLF. Five of the six stages were mutually exclusive, and bacteria that did not meet the requirements of each stage were discarded.

### 3.1. Bacterial Diversity

A culture-dependent isolation was conducted to explore the potential of these bacterial strains. The colonies visually observed were randomly selected based on their morphological characteristics and then isolated. One hundred sixty-one bacterial colonies were isolated and used for further experimentation.

A catalase test was used as the first stage of the screening process to evaluate the ability of the 161 isolates to decompose hydrogen peroxide [21]. Twenty-three isolates identified as catalase-negative were selected to continue with phenotype identification. A Gram-staining was conducted as the second stage of the selection process, and only Gram-positive bacteria were selected for further experimentation. The 23 previously selected isolates were Gram-positive and moved on to the next stage. At this stage, the selected isolates were classified as LAB and identified at the molecular level through 16S rRNA gene amplicon sequencing.

**Figure 2:**
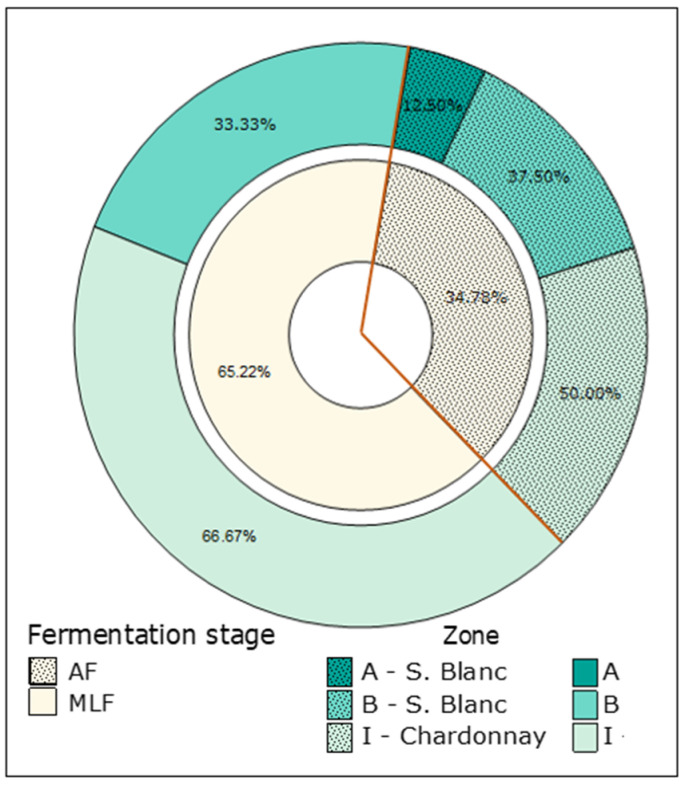
Distribution of isolated bacteria during spontaneous fermentation by fermentation stage and location at which grapes were harvested (A, B, and I). The S. Blanc grapes were collected from zones A and B, while Chardonnay grapes were from zone I.

Figure 2 illustrates the distribution of these isolates across the two fermentation stages (AF and MLF), categorized by grape harvest location. During AF, the proportion of isolated bacteria varied across locations A, B, and I. In contrast, during MLF, the majority of isolates originated from location I (66.66%), while none were obtained from location A. Additionally, a greater percentage of bacteria were isolated during MLF (65.22%) compared to AF (34.78%).

The Chardonnay cultivar in zone I yielded a significantly higher number of bacterial isolates compared to the Sauvignon Blanc in zones A and B during both AF and MLF. In contrast, Sauvignon Blanc from zone A had a low presence of culturable bacteria in both stages, resulting in the lowest percentage of isolates.

### 3.2. Bacterial Identification and Fermentative Metabolism

In this study, the third stage of the selection process involved assessing the fermentative metabolism and identifying the 23 selected isolates. The species, strain identification code, fermentation stage, zone, day, and cultivar from which the respective strains were isolated are shown in Table 2.

In this study, among the 23 selected isolates, two LAB species were identified. *Levilactobacillus brevis* (formerly *Lactobacillus brevis*) was the species with the highest abundance (61%), while *Leuconostoc mesenteroides* was found in a lower percentage (39%). According to Bergey et al. (1984) [22], all the LAB identified have been classified as heterofermentative bacteria. Thus, the fermentative metabolism of the selected isolates was determined by gas (CO_2_) formation and the final pH in the medium. Table 2 also shows the results obtained from the study of the fermentative metabolism conducted as a third stage of the selection process.

The experiment was conducted in a BSB medium with an initial pH of 5.525 ± 0.05 (color turning point). Among the isolates, those with lower fermentative capacity, such as BCV-37, BCV-47, and BCV-67, induced a pH reduction of only 0.5 units. In contrast, other LAB (for example BCV-156), achieved a more pronounced pH decrease of up to 1.2 units. For instance, for *L. brevis*, the species with the highest number of isolates, some strains caused only minor pH decreases, reaching values of 5 or slightly below. Meanwhile, other strains resulted in more significant acidification, lowering the pH to around 4.3. Finally, eight of the twenty-three selected LAB strains did not produce gas within the first 24 h of fermentation, suggesting a slower fermentative metabolism. This slower activity may promote enhanced secondary metabolic pathways and reduce the risk of volatile acidity increases during MLF.

To identify starter cultures suitable for MLF, we selected the isolates that exhibited the slowest fermentative metabolism, lacked gas production, and had low acidification. Eight LAB strains were chosen: six identified as *L. brevis* (BCV-37, BCV-40, BCV-42, BCV-46, BCV-48, and BCV-91), and the remaining two as *Leuc. mesenteroides* (BCV-39 and BCV-47).

### 3.3. Bacterial Growth in a Malic Acid-Enriched Medium

**Figure 3:**
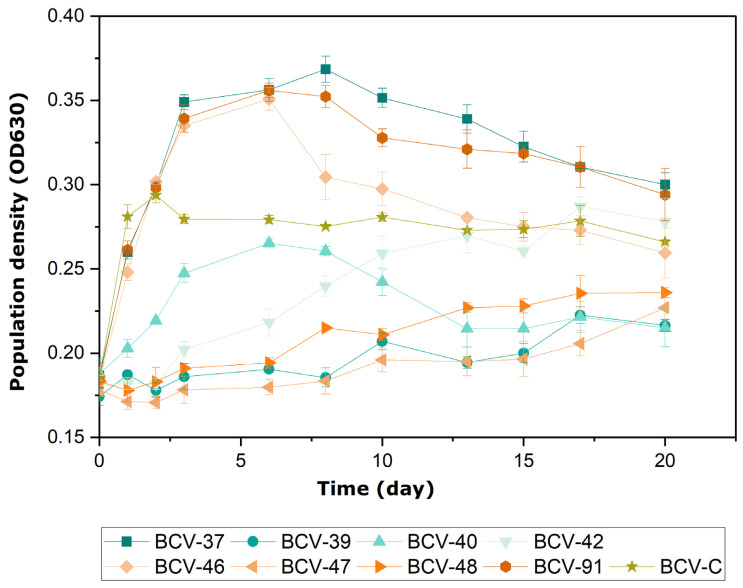
Evolution of lactic acid bacteria isolates’ growth in a malic acid-enriched test.

The fourth stage of the selection process consisted of evaluating the selected isolates’ ability to grow and perform MLF in a synthetic medium (M3m) with 10% *(v*/*v*) ethanol and pH 3.5. To test this ability, we investigated the growth behaviors of the eight selected LAB strains (Figure 3).

All selected isolates were able to grow in the enriched medium. However, the day at which each bacterium reached its growth peak was different. The control bacterium BCV-C (*L. plantarum*) was the fastest, achieving its growth peak in two days. The *L. brevis* strains (BCV-37, BCV-91, and BCV-46) expressed maximum growth within the first ten days of the experiment and then slowly declined in population. At the same time, higher populations were observed for these strains compared to the other five strains that displayed slower growth. For example, the strains *L. brevis* BCV-40 and BCV-42 showed a population density similar to the control bacterium BCV-C, although a sluggish growth response was observed. Similarly, the isolates BCV-48 (*L. brevis*), BCV-39 (*Leuc. mesenteroides*), and BCV-47 (*Leuc. mesenteroides*) exhibited even lower and slower growth than BCV-C, which might indicate that they were inhibited.

**Figure 4:**
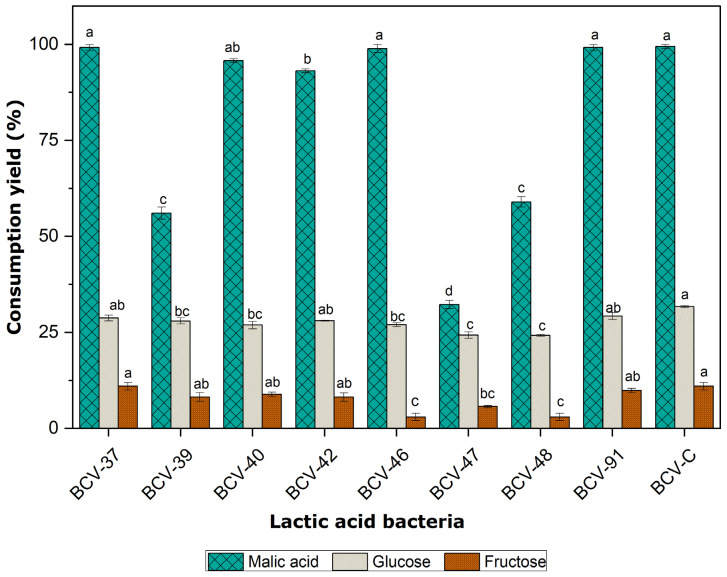
Malic acid, glucose, and fructose intake performance. Lower case letters represent significant differences within evaluated strains (*p* < 0.05). Separate statistical analyses were conducted for malic acid, glucose, and fructose consumption yields.

The consumption yields of L-malic acid, D-glucose, and D-fructose for each strain in the enriched medium are presented in Figure 4. The results were calculated based on the final concentration of each substrate. In this study, substrate consumption efficiency (consumption yield), expressed as a percentage, is defined as the difference between the initial and final concentrations of each independent substrate, divided by the initial concentration (as shown in Equations (3)–(5)).

Most bacteria consumed more than 30% of the L-malic acid in the medium. However, *L. brevis* strains BCV-37, BCV-46, and BCV-91 exhibited efficiencies comparable to the control strain BCV-C, consuming nearly 100% of the L-malic acid (with no statistically significant difference), positioning them as strong candidates for application as MLF starter cultures. These consumption yields correspond well with the growth profiles presented earlier (Figure 3), as strains with higher bacterial populations than the control BCV-C fully depleted the L-malic acid. Conversely, strains with lower growth, such as the *Leuc. mesenteroides* strains BCV-39 and BCV-47, and the *L. brevis* strain BCV-48, only partially consumed L-malic acid (56.084 ± 1.587%, 32.275 ± 1.058%, and 58.994 ± 1.322%, respectively).

Detected consumption yields were below 30% for glucose and 12% for fructose. Glucose was the preferred carbon source among the sugars, where the commercial control bacterium BCV-C showed the highest yield, while BCV-47 and BCV-48 had the lowest. Both BCV-37 and BCV-C exhibited the highest fructose consumption yields, with no significant differences between them.

Finally, each bacterium’s final lactic acid concentration was measured (Table 3)**.** Since this acid could be produced from consuming malic acid or sugars in the medium, the L-lactic acid percentage generated from malic acid was determined, and the difference was considered a product of sugar metabolism.

As it can be noted, the strains *L. brevis* BCV-37 and BCV-46 produced the highest concentrations of lactic acid (with no significant differences between them), followed by the strain *L. brevis* BCV-91. The concentration of L-lactic acid is related to the yields shown in Figure 4 since bacteria that meet or exceed the theoretically predicted concentration of lactic acid, determined by stoichiometric ratios, are the ones that achieve the most efficient malic acid and sugar consumption. The bacteria with low lactic acid production are the same bacteria that had a low malolactic yield and poor growth rates. The control bacteria BCV-C, *L. plantarum* ML Prime, had a high yield of lactic acid produced by malic acid consumption but a low lactic acid concentration produced by sugar consumption. BCV-C was designed to deliver a very high malolactic activity, and its facultative metabolism reduces the risk of volatile acidity production [23].

A significant competitive performance was achieved by BCV-37, BCV-46, and BCV-91, producing equal or higher lactic acid concentrations than the control bacterium. Based on the performance, these isolates were selected to test their survival under stress conditions.

### 3.4. Stress Tolerance Assessment

**Figure 5:**
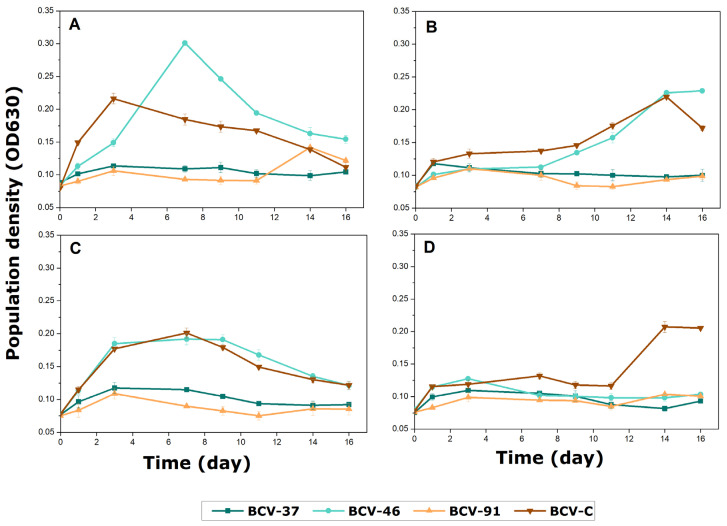
Resistance through time of lactic acid bacteria isolates under combined stress factors. Figure (**A**) represents Trial A (pH 3.5, Total SO_2_: 10 mg/L, ethanol: 11.5%), figure (**B**) represents Trial B (pH 3.5, Total SO_2_: 20 mg/L, ethanol: 13.5%), figure (**C**) represents Trial C (pH 3.2, Total SO_2_: 10 mg/L, ethanol: 11.5%), and figure (**D**) represents Trial D (pH 3.2, Total SO_2_: 20 mg/L, ethanol: 13.5%).

To assess stress tolerance, the selected bacteria were subjected to an assay involving three stress factors combined at two levels (Table 1) in a defined medium over a 16-day fermentation period. The resistance of the selected LAB strains over time is illustrated in Figure 5.

The inhibitory effect of these factors on the bacteria growth was evidenced from the start of the fermentation. This effect was more evident in the bacteria BCV-37 and BCV-91 since their growth was inhibited entirely in all tests. In contrast, the BCV-46 exhibited different behavior in all conditions.

In Trial A, maximum growth was achieved within the first seven days of the experiment. Although the peak occurred four days later than in the control bacterium (BCV-C), the cell concentration was significantly higher, reaching an OD630 of 0.30. Afterward, the bacterial concentration decreased, eventually reaching an OD630 of approximately 0.15, which was still higher than that of the control. In Trial C, BCV-46 exhibited similar behavior to BCV-C, with both reaching a peak concentration of around OD630 0.19. A subsequent decrease in cell density followed the same pattern, with OD630 falling below 0.15 by the end of the experiment.

In contrast, in Trial B, BCV-46 and the control BCV-C displayed an adaptive period, as indicated by a lag phase of up to seven days, followed by slow growth until the end of the tests. In Trial D, BCV-C followed a similar pattern, while BCV-46 was inhibited under these conditions. The results of Trial D showed that pH 3.2, a sulfur dioxide concentration of 20 mg/L, and 13.5% *(v*/*v*) ethanol were the most selective conditions.

Finally, in this study, *L. brevis* (BCV-46) showed the best performance at pH 3.5, total SO_2_: 10 mg/L, and ethanol: 11.5%, suggesting its potential as a starter culture for MLF in white wines.

### 3.5. Tyramine Production and Citric Acid Intake

To address both the health risks associated with tyramine production and the sensory impact of citric acid metabolism, we developed modified media tailored to each parameter. On one hand, a medium with tyrosine as the sole amino acid source was designed, and on the other, a defined medium was developed to evaluate citric acid metabolism. This dual approach aims to assess *L. brevis* strains for their potential as candidates for MLF starter cultures.

Tyramine production and citric acid intake were screened in the selected bacteria (BCV-37, BCV-46, BCV-91, and control BCV-C), with results shown in Table 4. Regarding tyramine production, all the strains were able to produce tyramine, as indicated by the green coloration surrounding the colonies. This outcome was expected, as the species of the selected bacteria have been reported as biogenic amine producers.

In contrast, citric acid consumption was positive only for the control bacterium, while the selected strains apparently did not consume this organic acid (Table 4). A clear and consistent color change in the colony was the primary criterion for identifying strains that tested positive for citric acid consumption.

## 4. Discussion

Diverse bacterial strains with genetic variation exist naturally on grape surfaces. During spontaneous AF and MLF, aliquots were aseptically collected and plated onto de Man, Rogosa, and Sharpe (MRS) agar for culture-dependent isolation. A total of 161 bacterial colonies were isolated, 23 of which exhibited morphological characteristics consistent with LAB. The main culture media used to isolate lactic acid bacteria are Rogosa medium and MRS medium [24]. These selective media are guaranteed to restrict the growth of other microorganisms. However, Matevosyan et al. (2019) [25] investigated various combinations (associations) of LAB strains in MRS medium, incorporating fungal and pathogenic bacterial strains in the experiments, all of which were capable of growing in this medium. Morphological characterization confirmed that the isolated strains were representative of the target bacterial group for this study.

LAB are conventionally associated with members of the genera *Lactobacillus*, *Leuconostoc*, *Pediococcus*, and *Oenococcus*, which are naturally present in grape must. These LAB have been characterized as Gram-positive and catalase-negative [22]. In our study, only 23 isolates met these characteristics which displayed significant diversity (Figure 2). Differences in wine microbial profiles during vinification have been associated with both endogenous and exogenous factors, including distinct cultivars, vintage, must features, and fermentation type [1,26,27]. Bubeck et al. (2020) [26] studied bacterial diversity and composition in red and white wines and reported that the variation in relative and absolute bacterial compositions was generally associated with AF (one-week fermentation).

The higher number of bacterial isolates in Chardonnay must may be due to the higher initial pH of the Chardonnay must (pH = 3.335 ± 0.024) compared to S. Blanc (pH = 3.105 ± 0.044), which could influence bacterial populations. In the study conducted by Cinquanta et al. (2018) [28], MLF in white Falanghina wines was completed more rapidly at pH levels of 3.4 and 3.8 when inoculated with *Oenococcus oeni*. In contrast, no MLF occurred at pH 3.2, suggesting that the lower pH inhibited LAB activity and population growth. Similarly, the study of Sun et al. (2018) [29] reported the influences of pH on the survival capacity of two LAB species where a pH value of 3.2 was detrimental to the experiment.

Furthermore, in this study, the fermentation of the Chardonnay must finished at a density close to 999 g/L and had a low alcohol content (5.693 ± 0.533%), which may have favored bacterial growth. On the other hand, S. Blanc from zones A and B completed fermentation (density < 993 g/L), with zone B having the highest alcohol concentration (11.360 ± 0.311%). It is well known that in climatic conditions, the pH of the must, the ethanol level, and the initial sulfiting influence the LAB development [11]. Additionally, yeasts release a wide range of metabolites, such as ethanol and certain fatty acids, hindering LAB growth during the AF process [30].

Exploring indigenous strains within specific regional or varietal conditions holds promise for discovering new strains characterized by enhanced genetic diversity and improved adaptation to local environments [31]. Studies have reported the LAB diversity in grape musts, including *L. plantarum*, *L. brevis*, *L. casei*, *P. damnosus*, *P. pentosaceus*, *Leuc. mesenteroides* and *O. oeni*, which are commonly isolated [10,15,32,33]. In this study, *L. brevis* and *Leuc. mesenteroides* were found in both cultivars (Table 2). These bacteria have been previously reported and isolated from spontaneous fermentations. Franco et al. (2021) [15] isolated LAB from different Chilean valleys and reported that the most abundant species was *Leuc. mesenteroides* found in nine grape varieties, including Chardonnay and S. Blanc. Prieto et al. (2007) [34] reported a relative frequency of 33% of *L. brevis* isolated from a Cabernet Sauvignon grapevine variety from the Aconcagua Valley, and *Leuc. mesenteroides* was detected with a frequency of 5% in six grape varieties including S. Blanc [35].

Although, to our knowledge, little information is available regarding the presence of *L. brevis* during wine fermentations, some studies have reported the isolation of this species in various regions and wine varieties. These include artisanal wineries in Italy during MLF [36]; five commercial wineries in the Western Cape, South Africa, during the 2001 and 2002 harvest seasons [37]; and fifty wine samples (six Chardonnay, twenty Spätburgunder, and twenty-four Weißburgunder) from experimental winemaking in Neustadt, Germany [38]. González-Arenzana et al. (2012) [9] isolated this bacterial species from different fermentations in the La Rioja region of Spain, where it was commonly found at the onset of AF. Similarly, Hu et al. (2022) [39] studied an autochthonous *L. brevis* isolated from early stages of an AF as a potential starter culture in co-culture fermentation with yeast. In this study, three *L. brevis* strains were isolated from the AF, while the majority were isolated from the MLF, suggesting that these bacteria could potentially be able to withstand the changes that occurred in the AF and pass to the MLF. However, further testing is required to confirm that the isolated bacteria are suitable starter cultures for MLF.

The heterofermentative *Oenococcus*, *Leuconostoc,* and *Lactobacillus* spp. lack EMP pathway enzymes, fermenting hexose sugars through the phosphoketolase pathway. An equimolar lactic acid, CO_2_, and acetic acid/ethanol from one glucose mole are released, of which lactic acid is the one that is produced in a more significant proportion (>50%) [40,41]. From this pathway, important traits for the metabolic characterization of heterofermentative LAB can be identified: the release of CO_2_ as a result of glucose consumption, an increase in acidity in the medium, and a decrease in pH due to acid production [42]

According to the results of the third stage of the selection process, the changes in pH were highly variable and did not exhibit a consistent correlation with the species of each strain (Table 2). The fermentative metabolism, following similar colorimetric methodology, has been studied by Hocine et al. (2010) [43]. The experiment was conducted for two weeks, and twenty-four *O. oeni* strains were identified as heterofermentative without false positive, so this study recommended this method to facilitate the comparison of numerous *O. oeni* strains. Additionally, the study by Du Plessis et al. (2004) [44] identified thirty-seven heterofermentative LAB in different fermentation stages, including *L. brevis*, which grew slowly in an incubation period of 2 to 7 days. Slow growth could be associated with the strain, which ferments only small amounts of sugar and tends to grow better in the presence of fructose [11].

On the other hand, LAB strains exhibit strain-specific genetic variability in their response to environmental conditions, and each LAB can lead to atypical behaviors with significant strain dependence [31]. For example, the fermentation patterns of carbohydrates differ significantly among various *Leuconostoc* spp. and are frequently influenced by the specific strain [8]. Based on these results, in this study, it could be inferred that specific mutations or genetic variants altered the fermentative metabolism associated with the performance of each strain of the same species.

Bacterial population growth in wine is affected by several physical–chemical factors, such as temperature, pH, alcohol, sulfur dioxide (SO_2_), nutrient limitation, or other potential as yet unknown factors [5]. The main stressor in wine is ethanol, and the LAB tolerance has been associated with the control of membrane integrity and the bacteria’s stress response during fermentation. Additionally, the expression of malolactic enzymes has been indirectly related to the increase of ethanol resistance [45,46]. Ethanol influenced the growth and malolactic activity of the selected bacteria differently during the fourth stage of the selection process. For example, BCV-37 showed growth similar to the control strain BCV-C, while BCV-47 was inhibited (Figure 3). The degree of ethanol tolerance varies according to the ability of each strain to grow and survive, which is influenced by environmental factors. However, growth inhibition rises as the alcohol concentration increases above 10% (*v*/*v*) [23]. Also, strains of *L. casei* and *L. brevis* have been described to be more tolerant and have successfully induced MLF in harsh conditions [47].

This pattern was consistent with the results obtained in the consumption yield of L-malic acid, D-glucose, and D-fructose for each strain in the enriched medium (Figure 4). The success of malic acid consumption is highly strain-dependent and can be influenced by several factors, including alcohol and sulfur dioxide concentrations, pH levels below 4.5, and limited nutrients [3,31]

In the study undertaken by Sun et al. (2016) [18], native LAB isolated from Cheery wine were selected after a similar selection procedure to the one performed in this study, where LAB were tested in a synthetic wine with 10% (*v*/*v*) ethanol. During 45 days of the experimentation, the authors found that most of the tested strains were able to consume all the L-malic acid; however, only 16 strains were selected because of their faster growth within 20 days, as in this study. Few researchers have reported the efficiency of malic acid consumption by *L. brevis* as a potential culture to drive wine MLF, and no study, to our knowledge, has focused on the study of *Leuc. mesenteroides*. In the study of Jung and Lovitt (2010) [48], *L. brevis* had a high bioconversion rate of malic acid in green cider and defined growth media at 28 °C. Comparatively, a native strain of *L. brevis* achieved a growth of 2.5 OD and a consumption rate of L-malic acid of 87% when it was evaluated in a modified MRS at 25 °C [49]. In the case of *L. mesenteroides*, a 93% yield of malic acid consumption was reported for cider production with an alcohol content of 10% and a lactic acid concentration of 3.50 g/L at the end of MLF [50].

When the wine pH is lower than 4.5, the ability to obtain energy from glucose is less efficient, and the bacterium reduces its growth in order to maintain the internal pH [47]. Under these conditions, *Lactobacillus* spp. can activate alternative metabolic pathways, such as malolactic fermentation; citrate, amino acid, and polyol metabolism; and the synthesis and hydrolysis of esters. These pathways play a crucial role in shaping the aroma and flavor profile of wine [51]. Overall, the fructose consumption rate was lower than expected, which is advantageous since mannitol, a byproduct of fructose metabolism [52], is considered a bacterial spoilage product. The findings of this study indicate that glucose and fructose served as secondary substrates for all the studied bacteria.

The strains *Lactobacillus brevis* BCV-37, BCV-46, and BCV-91 produced equal or higher final lactic acid concentrations than those of the control strain BCV-C. Lactic acid is the primary product of MLF, where malic acid-utilizing bacteria follow a well-defined mechanism: First, they actively transport malate from the extracellular environment into the cytoplasm via a specific malate–permease system. Second, these bacteria synthesize a cytoplasmic malolactic enzyme, which catalyzes the decarboxylation of malate to lactate. Finally, L-lactic acid is exported into the extracellular medium as a reaction product [2]. This mechanism establishes a direct stoichiometric relationship between malic acid consumption and lactic acid production, allowing the assessment of each strain’s efficiency in both primary and secondary metabolism (Table 3).

The *Lactobacillus* genus has been explored as an alternative to MLF performances. Lucio et al. (2016) [53] studied thirty-one strains *(Lactobacillus mali, Lactobacillus pantheris, Lactobacillus paracasei, Lactobacillus plantarum, Lactobacillus satsumensis*, *and Lactobacillus vini)* to evaluate the ability to carry out MLF in MC broth (medium to simulate white grape must). They demonstrated that most of the strains studied obtained a yield of 100% malic acid consumption and high lactic acid synthesis. The study of López-Seijas et al. (2020) [7] confirmed that malolactic activity is a property of the strain and not of the species by evaluating the enological characteristics of sixteen native *Lactobacillus* strains. A malic acid consumption yield between 0 to 78% and lactic acid production of up to 4 g/L was displayed, demonstrating that MLF depends on the strain that conducts it.

There is substantial evidence of strain-specific differences in response to stress conditions from the onset of fermentation. Ethanol is often the primary stressor in wine, followed closely by low pH levels. Additionally, sulfur dioxide is another element considered a stressor factor, due to the antimicrobial effect [5]. Most wines have a concentration ranging from 11.5% to 13.5% ethanol and a pH ranging from 3.2 to 3.65, while sulfur dioxide concentrations vary depending on vinification practices. When combined, these stressors, especially pH and SO_2_, have been shown to have a synergistic effect [54,55]. Thus, the isolates were assessed under stress conditions (Figure 5). These results are in agreement with those obtained by Sun et al. (2016) [18]. The data collected suggest that strains increase their population under more variable pH conditions (Trials A and C); however, limited growth became more evident when the ethanol and SO_2_ concentrations increased, as previously found by Guzzon et al. (2009) [56]. Similar to Trials B and D, a prolonged lag phase at the lowest pH or high SO_2_ presence was observed in the study conducted by Guzzon et al. (2009) [56].

Many studies have evaluated the tolerance of the genus *Lactobacillus* to different factors in wine. However, to our knowledge, this is the first study to evaluate the stress response of *L. brevis*; nevertheless, other LAB strains have been studied. For instances, Bravo-Ferrada et al. (2013) [57] demonstrated that *O. oeni* and *L. plantarum* exhibited lower relative growth rates under SO_2_ concentrations ranging from 50 to 500 mg/L at low pH (3.5), identifying SO_2_ as a key factor limiting growth. Similarly, Miller et al. (2011) [58] observed significant variability in the malolactic expression rate among different *L. plantarum* strains when subjected to high ethanol content (up to 15%) and low pH (3.2–3.8). Their findings indicate that the expression of the *mle* gene in *L. plantarum* strains was intensified at low pH values and reduced in the presence of ethanol. Additionally, a native Chilean *O. oeni* strain was identified by Romero et al. (2018) [59] as the best strain based on its technological properties, demonstrating the highest survival rate at 12–15% ethanol, high pH tolerance (3.1–3.6), and high viable count over ten days under the influence of sulfur dioxide (0–80 mg/L).

*Lactobacillus* has conventionally been associated with two important limitations, especially when evaluating them as potential starter cultures. These limitations involve their capacity to produce biogenic amines and/or undesirable aromas during citrate fermentation. Biogenic amines, such as histamine, tyramine, and putrescine, are formed through the decarboxylation of amino acids, and their presence in wine can raise health concerns [11]. *L. brevis* strains have been associated to tyramine formation in wine [60,61], and tyramine production has been identified as a species-level trait [62].

Previous studies have described amino acid decarboxylation in *L. brevis* [53,63,64] and *L. plantarum* [28,53,57], influenced by factors such as low pH, precursor availability, and gene expression. For example, tyrosine decarboxylase expression has been observed in the *L. brevis* IOEB 9809 strain [60,65]. M. V. Moreno-Arribas et al. (2003) [66] evaluated different LAB strains isolated from wine, where *L. brevis* demonstrated the ability to produce tyramine in modified decarboxylase media. Landete et al. (2007) [63] further confirmed that tyramine production is a common trait of *L. brevis*, with all tested strains producing this amine in synthetic media and wine. The experiments conducted by Arena et al. (2011) [60] indicated that *L. brevis* IOEB 9809 is able to produce both tyramine and putrescine during wine fermentation. However, while this study concluded that the selected strains produce tyramine, quantitative analysis is still needed to assess their full capacity to produce this biogenic amine in wine.

Additionally, citric acid, naturally present in must at low concentrations, is metabolized during fermentation, leading to the formation of compounds with sensory relevance, such as acetate, diacetyl, and other volatiles [13]. The lack of citrate fermentation detection could be attributed to heterofermentative bacteria assimilating this compound more slowly than malic acid, limiting production of acetoinic compounds, acetic acid, and fatty acids required for lipid synthesis [11]. As a result, the lack of color change in the qualitative medium for the native strains is likely due to their slower metabolism of citric acid, rather than to the absence of its consumption. Nevertheless, further studies are necessary to accurately detect and quantify citric acid uptake.

A study by Cinquanta et al. (2018) [28] demonstrated that the formation and concentration of acetic acid in citric acid metabolism is enhanced under higher pH conditions and varies among strains. The distinctive fermentation characteristics among seven strains of *L. brevis* were primarily determined by their capacities to metabolize sucrose and citric acid [67]. Under the applied experimental conditions, all *L. plantarum* isolates exhibited positive results for citrate utilization [57]. Finally, later studies on *L. brevis* showed that these features depend on the strain and vary in their ability to produce amines from amino acids and to utilize citrate [67].

## 5. Conclusions

Malolactic fermentation is a critical process in the production of Chardonnay wines, primarily driven by lactic acid bacteria, with *Oenococcus oeni* being the most commonly used species. In recent years, other naturally occurring lactic acid bacteria in grapes and vineyard environments have been investigated as potential starter cultures for MLF. In this study, bacteria were isolated from spontaneous fermentations of Chardonnay and Sauvignon Blanc, identifying species from the genera *Leuconostoc* and *Lactobacillus*. Among these isolates, BCV-46 (*Levilactobacillus brevis*) was identified as a strong candidate due to its high efficiency in converting malic acid to lactic acid. This isolate also demonstrated notable resistance to ethanol (10–13.5%), sulfur dioxide (20 mg/L), and low pH (3.2–3.5), suggesting its potential as a viable MLF starter culture. However, BCV-46 was also found to potentially produce tyramine, an undesirable trait for MLF starter cultures. A negative result for citrate consumption was observed in a qualitative assay. Further research is necessary to fully evaluate this isolate’s suitability for winemaking and its impact on the chemical and volatile profiles of the wine

## Figures and Tables

**Table 1:** Combined stress factors.

Trial	Stressor Factors	Level
A	pH:	3.5
	Total SO_2_ (mg/L):	10
	Ethanol (%):	11.5
B	pH:	3.5
	Total SO_2_ (mg/L):	20
	Ethanol (%):	13.5
C	pH:	3.2
	Total SO_2_ (mg/L):	10
	Ethanol (%):	11.5
D	pH:	3.2
	Total SO_2_ (mg/L):	20
	Ethanol (%):	13.5

**Table 2:** Molecular 16S rRNA identification of the isolates and fermentative metabolism.

Strain ^1^	Stage ^2^	Zone ^3^	Day	Cultivar	16S rDNA Gene Partial Sequences	GenBank Accession Number	Carbohydrate Metabolisms After 24 h Fermentation ${pH}_{BSB}=5.525\pm 0.05^{a}$	
							Gas Formation ^4^	pH
BCV-37	AF	I	2	Chardonnay	*Levilactobacillus brevis*	OR395097	−	5.025 ± 0.050 ^b^
BCV-39	AF	I	2	Chardonnay	*Leuconostoc mesenteroides*	OR395098	−	4.725 ± 0.050 ^c^
BCV-40	AF	I	2	Chardonnay	*Levilactobacillus brevis*	OR395099	−	4.525 ± 0.050 ^d^
BCV-42	AF	I	2	Chardonnay	*Levilactobacillus brevis*	OR395100	−	4.625 ± 0.050 ^cd^
BCV-46	AF	B	9	S. Blanc	*Levilactobacillus brevis*	OR395101	−	4.725 ± 0.050 ^c^
BCV-47	AF	B	9	S. Blanc	*Leuconostoc mesenteroides*	OR395102	−	5.025 ± 0.050 ^b^
BCV-48	AF	B	9	S. Blanc	*Levilactobacillus brevis*	OR395103	−	4.725 ± 0.050 ^c^
BCV-67	AF	A	4	S. Blanc	*Leuconostoc mesenteroides*	OR395104	+	5.025 ± 0.050 ^b^
BCV-85	MLF	I	48	Chardonnay	*Leuconostoc mesenteroides*	OR395105	+	4.525 ± 0.050 ^d^
BCV-86	MLF	I	48	Chardonnay	*Leuconostoc mesenteroides*	OR395106	+	4.525 ± 0.050 ^d^
BCV-87	MLF	I	48	Chardonnay	*Leuconostoc mesenteroides*	OR395107	+	4.525 ± 0.050 ^d^
BCV-91	MLF	B	48	S. Blanc	*Levilactobacillus brevis*	OR395108	−	4.725 ± 0.050 ^c^
BCV-94	MLF	I	48	Chardonnay	*Levilactobacillus brevis*	OR395109	+	4.325 ± 0.050 ^e^
BCV-97	MLF	B	48	S. Blanc	*Leuconostoc mesenteroides*	OR395110	+	4.525 ± 0.050 ^d^
BCV-101	MLF	B	48	S. Blanc	*Levilactobacillus brevis*	OR395111	+	4.525 ± 0.050 ^d^
BCV-102	MLF	B	48	S. Blanc	*Levilactobacillus brevis*	OR395112	+	4.625 ± 0.050 ^cd^
BCV-105	MLF	I	48	Chardonnay	*Levilactobacillus brevis*	OR395113	+	4.325 ± 0.050 ^e^
BCV-109	MLF	I	48	Chardonnay	*Levilactobacillus brevis*	OR395114	+	4.325 ± 0.050 ^e^
BCV-110	MLF	I	48	Chardonnay	*Leuconostoc mesenteroides*	OR395115	+	4.325 ± 0.050 ^e^
BCV-113	MLF	I	48	Chardonnay	*Levilactobacillus brevis*	OR395116	+	4.325 ± 0.050 ^e^
BCV-135	MLF	I	48	Chardonnay	*Levilactobacillus brevis*	OR395117	+	4.325 ± 0.050 ^e^
BCV-156	MLF	B	46	S. Blanc	*Levilactobacillus brevis*	OR395118	+	4.325 ± 0.050 ^e^
BCV-159	MLF	I	48	Chardonnay	*Leuconostoc mesenteroides*	OR395119	+	4.325 ± 0.050 ^e^

Lower case letters represent significant differences within the columns (*p* < 0.05). ^1^ Strain identification code: a unique identification code for each strain isolated consisting of letters and numbers. ^2^ Stage: AF: alcoholic Fermentation; MLF: malolactic fermentation. ^3^ Zone: three different geographic zones (Chardonnay from zone I and S. Blanc from zones A and B). ^4^ Gas formation: (+) positive or (−) negative CO_2_ production, indicated by gas accumulation in the Durham vials. ${pH}_{BSB}$ represents the pH of the sugar broth medium (SBS).

**Table 3:** Final lactic acid concentration and yield of lactic acid produced from malic acid and sugar.

Strain	Identification	$L_{T}$ (g/L)	${\%R}_{L\to M}$	${\%R}_{L\to S}$
BCV-37	*L. brevis*	1.835 ± 0.005 ^a^	68.642 ± 0.362 ^d^	31.357 ± 0.362 ^a^
BCV-39	*Leuc. mesenteroides*	0.900 ± 0.030 ^f^	79.134 ± 0.398 ^bc^	20.865 ± 0.398 ^bc^
BCV-40	*L. brevis*	1.665 ± 0.025 ^c^	73.039 ± 0.693 ^cd^	26.96 ± 0.693 ^ab^
BCV-42	*L. brevis*	1.510 ± 0.050 ^d^	78.372 ± 2.150 ^bc^	21.627 ± 2.15 ^bc^
BCV-46	*L. brevis*	1.820 ± 0.020 ^ab^	69.041 ± 1.496 ^d^	30.959 ± 1.496 ^a^
BCV-47	*Leuc. mesenteroides*	0.510 ± 0.010 ^g^	80.330 ± 1.059 ^b^	19.669 ± 1.059 ^c^
BCV-48	*L. brevis*	0.940 ± 0.020 ^f^	79.683 ± 0.091 ^bc^	20.316 ± 0.091 ^bc^
BCV-91	*L. brevis*	1.675 ± 0.035 ^bc^	75.245 ± 2.173 ^bcd^	24.754 ± 2.173 ^abc^
BCV-C	*L. plantarum*	1.260 ± 0.001 ^e^	99.181 ± 0.264 ^a^	0.818 ± 0.264 ^d^

$L_{T}$: is the final concentration of lactic acid in the medium (g/L). ${\%R}_{L\to M}$ and ${\%R}_{L\to S}$ are the yields of lactic acid coming from MLF and sugar fermentation (%), respectively. Lower case letters represent significant differences within the columns (*p* < 0.05).

**Table 4:** Tyramine production and citric acid consumption response.

Strain	Tyramine Response		Citric Acid Response	
	Reaction	Modified Decarboxylating Agar Result	Reaction	Differential Medium Agar Result
BCV-37	Positive	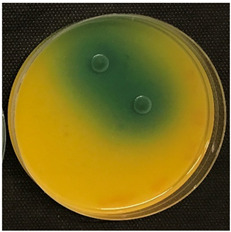	Negative	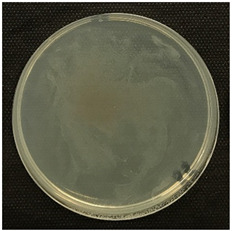
BCV-46	Positive	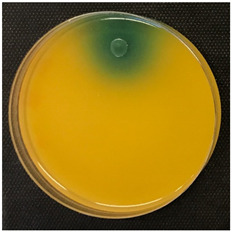	Negative	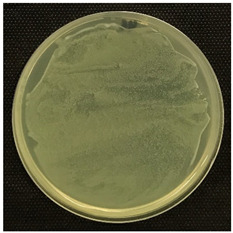
BCV-91	Positive	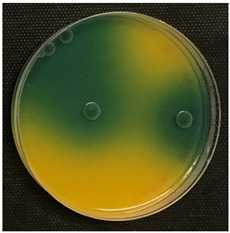	Negative	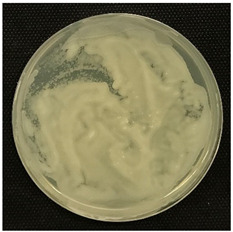
BCV-C	Positive	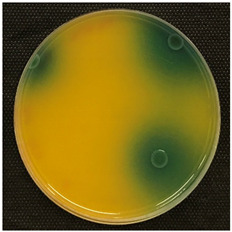	Positive	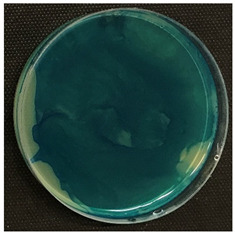

## Data Availability

The original contributions presented in the study are included in the article, further inquiries can be directed to the corresponding author.
